# Migrant tuberculosis: the extent of transmission in a low burden country

**DOI:** 10.1186/1471-2334-12-60

**Published:** 2012-03-18

**Authors:** Zaza Kamper-Jørgensen, Aase Bengaard Andersen, Axel Kok-Jensen, Mads Kamper-Jørgensen, Ib Christian Bygbjerg, Peter Henrik Andersen, Vibeke Østergaard Thomsen, Troels Lillebaek

**Affiliations:** 1 International Reference Laboratory of Mycobacteriology, Statens Serum Institut, Copenhagen, Denmark; 2 Department of Infectious Diseases, Odense University Hospital, Odense, Denmark; 3 Department of Public Health, University of Copenhagen, Copenhagen, Denmark; 4 International Health Unit, Department of International Health, Immunology and Microbiology, University of Copenhagen, Copenhagen, Denmark; 5 Department of Epidemiology, Statens Serum Institut, Copenhagen, Denmark; 6 Department of Infectious Diseases, Rigshospitalet, Copenhagen University Hospital, Copenhagen, Denmark; 7 International Reference Laboratory of Mycobacteriology, Statens Serum Institut, 5 Orestads Boulevard, DK-2300 Copenhagen, Denmark

**Keywords:** Denmark, Genotyping, IS*6110*-RFLP, Molecular epidemiology

## Abstract

**Background:**

Human migration caused by political unrest, wars and poverty is a major topic in international health. Infectious diseases like tuberculosis follow their host, with potential impact on both the migrants and the population in the recipient countries. In this study, we evaluate *Mycobacterium tuberculosis *transmission between the national population and migrants in Denmark.

**Methods:**

Register study based on IS*6110*-RFLP results from nationwide genotyping of tuberculosis cases during 1992 through 2004. Cases with 100% identical genotypes were defined as clustered and part of a transmission chain. Origin of clusters involving both Danes and migrants was defined as Danish/migrant/uncertain. Subsequently, the proportion of cases likely infected by the "opposite" ethnic group was estimated.

**Results:**

4,631 cases were included, representing 99% of culture confirmed cases during 1992 through 2004. Migrants contributed 61.6% of cases. Up to 7.9% (95% CI 7.0-8.9) of migrants were infected by Danes. The corresponding figure was 5.8% (95% CI 4.8-7.0) for Danes. Thus, transmission from Danes to migrants occurred up to 2.5 (95% CI 1.8-3.5) times more frequent than vice versa (OR = 1). A dominant strain, Cluster-2, was almost exclusively found in Danes, particular younger-middle-aged males.

**Conclusions:**

Transmission between Danes and migrants is limited, and risk of being infected by the "opposite" ethnic group is highest for migrants. TB-control efforts should focus on continues micro-epidemics, e.g. with Cluster-2 in Danes, prevention of reactivation TB in high-risk migrants, and outbreaks in socially marginalized migrants, such as Somalis and Greenlanders. Fears that TB in migrants poses a threat for resident Danes seem exaggerated and unjustified. We believe this to be true for other low incidence countries as well.

## Background

Human migration caused by political unrest, wars and poverty is a major topic in international health. Infectious diseases like tuberculosis (TB) follow their host, with potential impact on both the migrants and the population in the recipient countries. The strict health regulations in relation to migration implemented by some TB low burden countries could imply, that the population in the receiving countries is at high risk of contracting TB from migrants, a perception sometimes promoted by politicians and parts of the media [1]. The present study was conducted in Denmark (DK), a wealthy and developed country of 5.5 million inhabitants [2], with low TB incidence (7.2/100,000 population/year) [3]. We analysed the impact of migration from TB high burden countries to DK on TB transmission, based on results of nationwide routine genotyping of *Mycobacterium tuberculosis *(MTB) strains from all culture verified TB cases during 1992 through 2004. Due to the wide geographical coverage of genotyping and the long observation period, resulting in a large number of cases, we were able to study both the likely direction of transmission and the extent of transmission between the national population and migrants.

## Material

The present case-only study is based on The Danish TB Notification Register (TBNR), The Danish *Mycobacterium tuberculosis *complex (MTBC) Genotyping Register and the Danish Civil Registration System (CRS).

### The Danish notification system

The Kingdom of DK comprises DK, and the autonomous countries Greenland and the Faeroe Islands. All countries have separate notification systems, but a common diagnostic facility, The International Reference Laboratory of Mycobacteriology (IRLM) at Statens Serum Institut (SSI) in Copenhagen, performing all culturing, drug susceptibility testing and molecular typing of MTBC strains. Microscopy services are also offered by regional microbiology- and pathology laboratories. The mandatory TB notification system is dual: physicians in hospitals as well as the IRLM must report new and recurrent TB cases to the Department of Epidemiology, SSI, and to the Danish National Board of Health. Further, TB diagnostics, treatment and contact tracing is free of charge for the individual, all factors enhancing surveillance quality.

### The Danish MTBC Genotyping Register

Since 1992, culture verified new and recurrent MTBC cases diagnosed within the Danish Kingdom have been genotyped routinely on a nationwide basis, and results stored in the *Mycobacterium tuberculosis *complex Genotyping Register. Genotyping results are linked to a unique civil registration number from the Danish CRS, and information on gender, country of birth, district of residence, date for and age at specimen collection, specimen, disease site, previous TB disease, laboratory findings (e.g. microscopy and drug susceptibility testing), hospital/clinic, and epidemiological linkage information, whenever available.

### Study cohort

The study cohort was retrieved from the *Mycobacterium tuberculosis *complex Genotyping Register, including only MTB cases from DK (not Greenland and the Faroe Islands), genotyped within the period 1992 through 2004. Each case was represented by one strain only, except for reinfection cases, defined by IS*6110- *Restriction Fragment Length Polymorphism (RFLP) patterns differing > 2 bands for each subsequent episode of cured/completed TB treatment. All relapse (≤ 2 bands), cross-contamination, *M. bovis*, and *M. bovis BCG *cases were excluded. Cases of *M. bovis *were excluded as the source of infection could be an animal, and as *M. bovis *disease is nearly eliminated in DK, with less than one imported case per year.

### Categorization and analysis

Ethnicity was based on country of birth registered in The *Mycobacterium tuberculosis *complex Genotyping Register, for the majority of cases retrieved via the Danish CRS. In DK, newborns are given a civil registration number by the CRS at birth. Migrants receive a temporary civil registration number until residence permit is granted. All cases without known country of birth had temporary civil registration numbers, and were therefore categorized as migrants. Cases born in Greenland, but diagnosed/treated in Denmark were categorized as migrants/Greenlanders.

The total cohort, including clustered and non-clustered cases, was stratified by sex and age (20-year age groups) (Table 1) and the probability of equal distribution of sex and age was tested using chi-square tests. The cohort was then stratified by genotype and sex (Table 2), according to ethnicity (Danes, Somalis, Greenlanders, "others" - pooling of remaining migrants). Mean age (MA) and standard deviation (SD) at time of diagnosis was then calculated.

**Table 1:** Genotyped cases, according to ethnicity, by age and sex

*n *(row %)*	Danes	Somalis	Greenlanders	Others **	Total	*p-value*
**Age**						*< 0.01 *

0-19 years	81 (15.1)	271 (50.7)	9 (1.7)	174 (32.5)	535 (100.0)	-

20-39 years	429 (20.1)	791 (37.9)	95 (4.6)	772 (37.0)	2,087 (100.0)	-

40-59 years	700 (58.4)	82 (6.8)	94 (7.8)	323 (26.9)	1,199 (100.0)	-

60-79 years	408 (69.5)	26 (4.4)	8 (1.4)	145 (24.7)	587 (100.0)	-

≥ 80 years	162 (89.5)	0 (0)	0 (0)	19 (10.5)	181 (100.0)	-

**Sex**						*< 0.01 *

Women	608 (31.5)	526 (27.3)	98 (5.1)	698 (36.2)	1,930 (100.0)	-

Men	1,172 (44.1)	644 (24.2)	108 (4.1)	735 (27.6)	2,659 (100.0)	-

All	1,780 (38.8)	1,170 (25.5)	206 (4.5)	1,433 (31.2)	* 4,589 (100.0)	

*Cases are represented by all *M. tuberculosis *strains included in study, genotyped by IS*6110*-RFLP/Spoligo in Denmark, 1992-2004, excluding cases without known country of birth (*n *= 42).

** Pooling of non-Somali-Greenlandic migrants

**Table 2:** Genotyped cases, according to ethnicity, by genotype and sex

*n *(column %)*	Danes	Migrants	Somalis	Greenlanders	Others**	Total
**High copy number clustered **(> 5 DNA bands)						

Males	842 (47.3)	610 (21.4)	299 (25.6)	92 (44.6)	219 (14.8)	1,452 (31.4)

Females	328 (18.4)	467 (16.4)	222 (19.0)	81 (39.3)	164 (11.1)	795 (17.2)

All	1,170 (65.7)	1,077 (37.8)	521 (44.6)	173 (83.9)	383 (25.9)	2,247 (48.5)

**Low copy number clustered **(≤ 5 DNA bands)						

Males	28 (1.6)	195 (6.8)	122 (10.4)	0 (0.0)	73 (5.0)	223 (4.8)

Females	9 (0.5)	198 (6.9)	117 (10.0)	1 (0.5)	80 (5.4)	207 (4.5)

All	37 (2.1)	393 (13.8)	239 (20.4)	1 (0.5)	153 (10.4)	430 (9.3)

**Non-clustered **(all band numbers)						

Males	302 (17.0)	709 (24.9)	223 (19.1)	16 (7.8)	470 (31.9)	1,011 (21.8)

Females	271 (15.2)	672 (23.6)	187 (15.9)	16 (7.8)	469 (31.8)	950 (20.5)

All	573 (32.2)	1,381 (48.4)	410 (35.0)	32 (15.6)	939 (63.7)	1,954 (42.2)

**All**	**1,780 (100.0)**	**2,851 (100.0)**	**1170 (100.0)**	**206 (100.0)**	**1475 (100.0)**	* 4,631

* Cases are represented by all *M. tuberculosis *strains included in study, genotyped by IS*6110*-RFLP/Spoligo in Denmark, 1992-2004, incl. cases without known country of birth (non-Danish) (*n *= 42).

** Pooling of non-Somali-Greenlandic migrants

The cohort of clustered cases was stratified by year, according to ethnicity (Table 3), and annual sex-related incidence rate (IR) (new cases/10^5 ^population/year) for clustered TB was estimated for the years 1992 through 2004, according to ethnicity (Table 4).

**Table 3:** Clustered cases, according to ethnicity, by year

Year *n *(row %)*	Danes	Migrants	Somalis	Greenlanders	Others **	Total
**1992**	86 (63.2)	50 (36.8)	13 (9.6)	5 (3.7)	32 (23.5)	136 (100.0)

**1993**	97 (53.3)	85 (46.7)	32 (17.6)	15 (8.2)	38 (20.9)	182 (100.0)

**1994**	107 (48.0)	116 (52)	61 (27.4)	5 (2.2)	50 (22.4)	223 (100.0)

**1995**	93 (43.5)	121 (56.5)	61 (28.5)	14 (6.5)	46 (21.5)	214 (100.0)

**1996**	101 (47.0)	114 (53.0)	63 (29.3)	9 (4.2)	42 (19.5)	215 (100.0)

**1997**	86 (34.5)	163 (65.5)	104 (41.8)	13 (5.2)	46 (18.5)	249 (100.0)

**1998**	112 (41.3)	159 (58.7)	90 (33.2)	15 (5.5)	54 (19.9)	271 (100.0)

**1999**	87 (35.7)	157 (64.3)	102 (41.8)	8 (3.3)	47 (19.3)	244 (100.0)

**2000**	94 (39.3)	145 (60.7)	91 (38.1)	13 (5.4)	41 (17.2)	239 (100.0)

**2001**	96 (46.8)	109 (53.2)	52 (25.4)	20 (9.8)	37 (18.0)	205 (100.0)

**2002**	73 (41.2)	104 (58.8)	37 (20.9)	23 (13.0)	44 (24.9)	177 (100.0)

**2003**	96 (56.1)	75 (43.9)	27 (15.8)	18 (10.5)	30 (17.5)	171 (100.0)

**2004**	79 (52.3)	72 (47.7)	27 (17.9)	16 (10.6)	29 (19.2)	151 (100.0)

**All**	1207 (45.1)	1470 (54.9)	760 (28.4)	174 (6.5)	536 (20.0)	* 2677 (100.0)

* Cases are represented by clustered *M. tuberculosis *strains included in study, high- and low copy number clustered patterns, genotyped by IS*6110*-RFLP/Spoligo in Denmark, 1992-2004, including cases without known country of birth (non-Danish, in "others") (*n *= 14).

** Pooling of non-Somali-Greenlandic migrants

**Table 4:** Incidence of clustered tuberculosis in Denmark 1992-2006, according to ethnicity, by age

n *		Danes		Migrants		Somalis		Greenlanders	Others**	Total		
**Age**	**♀**	**♂**	**Total**	**♀**	**♂**	**Total**	**Total**	**Total**	**Total**	**♀**	**♂**	**Total**

**0-19**	0,3	0,5	0,4	33,1	36,9	35,0	509,0	21,5	12,2	2,1	2,3	2,2

**20-39**	1,3	2,6	2,0	41,1	49,1	45,1	907,7	118,1	15,9	5,3	7,0	6,2

**40-59**	1,3	5,0	3,2	17,7	24,9	21,4	504,0	219,4	10,5	2,3	6,3	4,3

**60-79**	1,1	2,4	1,7	11,2	22,6	15,7	840,3	121,1	10,0	1,5	3,0	2,2

**80+**	1,3	2,4	1,6	3,3	10,1	4,9	0,0	0,0	5,0	1,3	2,6	1,7

**All**	**1,0**	**2,7**	**1,9**	**29,6**	**37,9**	**33,6**	**725,4**	**121,0**	**13,0**	**2,9**	**4,9**	**3,9**

* Cases are represented by clustered *M. tuberculosis *strains included in study (*n *= 2677), genotyped by IS*6110*-RFLP/Spoligo in Denmark, 1992-2004, including cases without known country of birth (non-Danish, in "others") (*n *= 14).

** Pooling of non-Somali-Greenlandic migrants

For estimation of transmission proportions (Table 5), only TB cases in mixed clusters (Danes/migrants) were considered likely MTB infected by the "opposite" ethnic group (numerator), but all genotyped TB cases (both clustered and non-clustered) were considered likely MTB infected (denominator). Both HCC and LCC genotypes (see Typing methods) were included in the transmission analysis.

**Table 5:** Proportion of TB transmitted with varying definition of mixed cluster origin

*	Likely origin of clusters (based on numbers of Danes/migrants in the individual cluster)								Proportion of TB cases likely due to transmission		Odds ratio
	**Danish** **(most Danes)**			**migrant** **(most migrants)**		**uncertain** **(even number)**					

**Ethnic composition** **of cluster**	**Mixed**		**Danish**	**Mixed**		**Migrant**	**Mixed**		**% (95% CI)**	**% (95% CI)**	**OR (95% CI)**

**Ehnicity of case**	**Migrants** ***n ***	**Danes** ***n ***	**Danes** ***n ***	**Migrants** ***n ***	**Danes** ***n ***	**Migrants** ***n ***	**Migrants** ***n ***	**Danes** ***n ***	**From migrants** **to Danes**	**From Danes** **to migrants**	

Origin of mixed clusters by criteria,											

Criteria 1 **	225	800	303	560	104	685	0	0	5.8 (CI 4.8-7.0)	7.9 (CI 7.0-8.9)	1.4 (CI 1.1-1.8)

Criteria 2 **	205	796	303	558	87	685	22	21	5.0 (CI 4.0-6.1)	7.3 (CI 6.4-8.3)	1.5 (CI 1.2-1.9)

Criteria 3 **	200	822	303	554	52	685	31	30	3.0 (CI 2.3-3.9)	7.1 (CI 6.2-8.1)	2.5 (CI 1.8-3.4)

Criteria 4 **	179	761	303	523	45	685	83	98	2.7 (CI 2.0-3.6)	6.5 (CI 5.6-7.5)	2.5 (CI 1.8-3.5)

Criteria 5 **	204	803	303	567	85	685	14	16	4.8 (CI 3.9-5.9)	7.2 (CI 6.3-8.2)	1.5 (CI 1.2-2.0)

* Cases are represented by clustered *M. tuberculosis *isolates, genotyped by IS*6110*-RFLP/Spoligo in Denmark, 1992-2004. Mixed clusters: *n = *105. Cases in mixed clusters: mmigrants: *n *= 785, Danes: *n *= 904.

** Criteria:

1: Origin of first patient in cluster defines cluster origin

2: Origin of first sputum smear positive patient in cluster defines cluster origin

3: Origin of most patients in cluster defines cluster origin

4: Origin of the 2/3 criteria met defines cluster origin

5: Origin of the 3/3 criteria met defines cluster origin

Clusters were categorized as being a either "mixed" (if comprised both Danes and migrants) or "Identical ethnicity cluster" (if comprised only Danes or migrants). "Identical ethnicity clusters" were further categorized into one of two cluster origin categories: "Danish" (if comprising Danish cases), or "migrant" (if comprising migrant cases), and cases within these two cluster origin categories were allocated according to ethnicity, in Danes or migrants. Mixed clusters were also categorized according to cluster origin, into one of three categories: "Danish" (if comprising predominantly Danish cases), "migrant" (if comprising predominantly migrant cases), or "uncertain" (if comprising an even number of Danes and migrants). As origin of a mixed cluster is more uncertain than a cluster containing only one nationality of cases, five different criteria were applied to define likely origin of the mixed clusters. We wanted to study if defining origin of mixed clusters in different ways would cause cases to distribute differently, altering transmission proportions. The five criteria used to define origin of a mixed cluster were: 1) ethnicity of first patient in the cluster; 2) ethnicity of first sputum smear positive patient in the cluster; 3) ethnicity of most patients in the cluster; 4) two of three criteria 1-3 meet; and 5) three of three criteria 1-3 met, defines the cluster origin. Cases within the three cluster categories were allocated according to ethnicity, in Danes or migrants.

The proportion of cases most likely transmitted from Danes was estimated, first including cases in mixed clusters of "uncertain" origin, then excluding cases in mixed clusters of "uncertain"origin. This was also done for migrants. The proportions in Table 5 are based on calculations excluding cases from mixed clusters of "uncertain" origin. The odds ratio (OR) of a Dane being infected by a migrant, compared with a migrant being infected by a Dane, within 95 percent confidence intervals (95%CI), was estimated for each of the five criteria, using logistic regression models. Data were processed and analysed in SAS version 9.1.

### Typing methods

During the study period 1992 through 2004, MTBC cases were genotyped by the IS*6110*-RFLP method [4], and isolates with low copy (LC) number patterns (≤ 5 bands) were further typed by spacer oligonucleotide (spoligo) typing [5], also identifying *M. bovis*. Identification of *M. bovis *BCG was done by IS*1081*-RFLP [6] and for early cases by thiopene-2-carboxylic acid hidrazide/pyrazinamide resistance pattern. Species identification to the level of MTBC was achieved by AccuProbe (Gen-Probe Incorporated) [7] or INNOLipa revers hybridisation assay (Innogenetics) [8]. Computer analysis was performed using the Bionumerics version 4.61 software (Applied Maths NV, Belgium). A high copy number cluster (HCC) was defined as ≥ 2 cases with 100% identical IS*6110*-RFLP genotypes and > 5 bands in the RFLP-pattern. A low copy number cluster (LCC) was defined as having a LC band RFLP-pattern and 100% identical spoligotypes.

## Results

The cohort consisted of 4,631 genotyped TB cases, representing 77% (23% of notified cases were culture negative or no specimen were sent for culture) of notified and 99% of culture verified cases in the study period. One percent of culture verified cases were non-typable.

### Cohort characterization

Of the 4631 cases, 45.8% originated from Europe (incl. Russia), 19.2% from Asia (incl. Turkey), 30.1% from Africa, 4.7% from North America (incl. Greenland), 0.2% from South America, and 42 had an unknown country of birth. Danes accounted for 38.8% (65.8% men) and migrants for 61.2% (52.9% men), distributed on Somalis 25.5% (55.0% men), Greenlanders 4.5% (52.4% men), and "others" 31.2% (51.3% men) (Table 1). In general, Nordic cases, excluding Greenlandic cases, were approximately twice as old (DK MA 51.2, SD 19.6) as African cases (Somalia (MA 27.4, SD 11)) at the time of diagnosis. Also, Asian and South American cases were younger (MA approx. 30), only Vietnamese, Afghan and Sri Lankan cases deviating (MA approx. 40). There were only few North American cases, other than cases from Greenland (MA 40.2, SD 10.6). MA for "other" cases was 36.7 (SD 16.8).

Clustered strains constituted 2677 cases, corresponding to 57.8% of all cases (Table 2), distributed into 403 clusters, 373 HCC and 30 LCC. Of these, 357 cases were < 20 years of age (68 Danes, 289 migrants), 1232 cases were between 20 to 39 years (359 Danes, 873 migrants), 802 cases were between 40 to 59 years (560 Danes, 242 migrants), 239 cases were between 60 to 79 years (177 Danes, 62 migrants) and 44 cases were 80+ years (40 Danes, 4 migrants). Clustered cases accounted for 67.8% of Danish and 51.6% of migrant cases. Of clustered cases, 90.7% Danes and 66.5% of migrants (97.1% of Greenlanders, 56.8% of Somalis and 70.3% of "others") had pulmonary TB.

Total IR of clustered TB was much higher in migrants (IR 33.6) than in Danes (IR 1.9), with peaks at age 20-39 years in migrants (IR 45.1) and at age 40-59 years (3.2) in Danes (IR 3.2) (Table 4). Particularly high total IR for clustered TB was observed in Somalis (IR 725.4) and Greenlanders (IR 121.0).

### Transmission between Danes and migrants

Of clusters, 26.1% (105/403) were mixed (Table 5), involving 904 Danes and 785 migrants. Of all mixed cases, 183 were children/adolescence < 20 years (53 Danes, 79 Somali, 3 Greenlandic) and 60 cases were children < 10 years (28 Danes, 23 Somali, 1 Greenlandic). About half of the mixed clusters (58/105), were identified within the first two years after implementation of MTBC genotyping in DK.

Depending on criteria applied to define mixed cluster origin, Danes accounted for 7.9% (45/568) to 15.7% (104/664) of cases in clusters of likely migrant origin, and migrants 19.0% (179/940) to 22.0% (225/1025) of cases in clusters of likely Danish origin. Including cases in mixed clusters of uncertain origin, 7.7% (95% CI 6.7-8.7) to 9.2% (95% CI 8.2-10.3) of migrants were most likely infected by Danes, and 4.6% (95% CI 3.7-5.7) to 8.0% (95% CI 6.8-9.4) of Danes were likely infected by migrants. Excluding cases in mixed clusters of uncertain origin from the estimation, 6.5% (95% CI 5.6-7.5) to 7.9% (95% CI 7.0-8.9) of migrants were most likely infected by Danes. For Danes, this proportion was between 2.7% (95% CI 2.0-3.6) and 5.8% (95% CI 4.8-7.0). Odds ratio of being infected by a person from "opposite" ethnic group was 1.4 (95% CI 1.1-1.8) to 2.5 (95% CI 1.8-3.5) times higher for migrants than for Danes (OR = 1), depending of criteria applied. Including these cases, odds ratio decreased, 1.2 (95% CI 0.9-1.4) to 1.8 (95% CI 1.4-2.4) for migrants. The findings were significant for both scenarios.

### Genotype distribution between ethnic groups

Greenlanders had the highest proportion of clustered strains, 84.5% (Table 2). Of these, 55 were found in the second largest cluster in DK designated "Cluster 1" (C-1), which is believed to originate from Greenland [9]. C-1 constituted 11.8% (142/1,207) of clustered cases in Danes, 2.6% (14/536) in "others" and < 0.1% (1/760) in Somalis. The largest cluster in DK, "Cluster 2" (C-2), constituted 26.3% (317/1,207) of clustered cases in Danes, 11.6% (20/174) in Greenlanders, 2.4% (13/536) in "others" and none in Somalis. A mixed cluster of likely Somali origin, LCC-3, constituted 14.3% (109/760) of clustered cases in Somalis, 13.2% (71/536) in "others", 0.4% (5/1,207) in Danes and none in Greenlanders. The overall proportion of LCC cases in migrants was 6.5 times that of Danes (Table 2).

**Figure 1:**
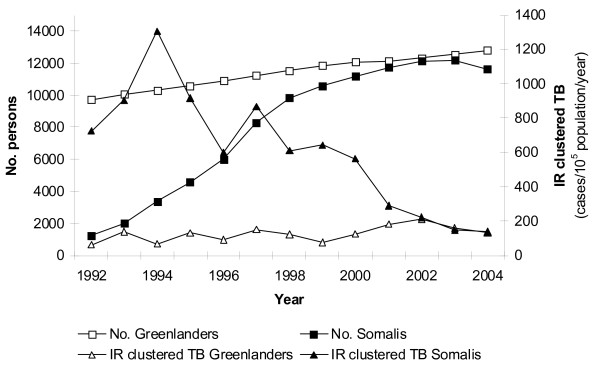
**Persons in Denmark and incidence of clustered tuberculosis, according to ethnicity, by time**.

For Somalis (Figure 1), the IR of clustered TB increased up to 1994, and decreased hereafter. In the same time period, the numbers of Somalis in DK increased, levelling of approximately year 2000. In comparison, IR of clustered TB and numbers of persons in DK were stabile or increased slightly for Greenlanders, at a much lower level. No decline in IR was observed in Greenlanders.

## Discussion

### Transmission between Danes and migrants in DK

In 1992 through 2004, 61.6% of all TB cases notified were migrant cases. One fourth of the 403 clusters included both migrants and Danes. In accordance with previous findings [10-15], we found only limited transmission between migrants and Danes. The proportion of TB cases likely transmitted by other ethnic groups was higher in migrants (up to 7.9% (95% CI 7.0-8.9)) than in Danes (up to 5.8% (95% CI 4.8-7.0)), with odds ratios up to 2.5 for migrants (Table 3). Limited transmission between migrants and the resident population has previously been shown for Somalis and Danes [13,14] in DK, and Mexicans and Americans in the US [13].

The limited transmission between migrants in DK and Danes is in part a result of poor integration [14], rather than an effective Danish migrant TB control strategy. Refugees in camps are only questioned about TB symptoms/screened at arrival, thus migrants only receive a very limited introduction to the Danish health care system. Also, active case finding within migrant TB high-risk groups has only been initiated on a minor basis the recent years.

Misclassification in registration of country of birth could confound the transmission proportion, as country of birth is a strong predictor for IR [16]. It is most likely that children born in DK by migrant parents would be misclassified as migrants. However, if corrected, this would only strengthen the association. Also, the contribution to transmission from the youngest of children is believed to be limited, as the numbers of minor migrant children in mixed clusters in this study were limited (32 cases < 10 years) and as minor children are mostly considered non-infective [16].

Categorization of clusters in Danish, migrant or uncertain origin is a simplification with many limitations. However, the use of other criteria, ideally based on detailed epidemiological linkage information, was not available. Therefore, to meet the uncertainty regarding origin of clusters, we analyzed the origin of mixed clusters by 5 different criteria, with largely the same result, supporting the association.

It must be expected that any group of migrants from TB high incidence countries, living for decades in a TB low incidence country, will gradually transmit some MTB strains to the resident population. However, our data indicate, that the exchange occurs at a low rate. Fears that TB in migrants poses a threat for resident Danes seem exaggerated and unjustified.

### Origin of migrant strains

In concordance with findings reported in a Dutch study among Somalis and Turks, where most recent transmission was attributable to transmission from cases with the same nationality [11], we also found limited transmission across ethnic subgroups. Even though some transmission has been confirmed by genotype and linkage information within/between ethnic subgroups in DK, in e.g. ethnic clubs, shelters, language schools, and the environment of homeless/socially marginalized, including khat-abusing Somalis in the capital region, clustering in migrants in DK more likely reflects reactivation disease [14]. Other studies has documented that clustering in migrants may reflect recent transmission in the recipient country [11,17], but that most cases are due to reactivation [14,18-22].

Greenlanders had the highest proportion of clustered strains (Table 2). This is not surprising, as clustering, like in the Somalis, likely reflects imported reactivation disease, and the Greenlandic population in Greenland is relatively isolated, with only few introductions of new MTB genotypes into the population. The TB incidence [23] is high, with a notification rate (NR) at approximately 150. In DK, many micro epidemics involving Greenlanders are seen in larger cities and the capital area, often involving the two largest clusters in DK, C-1 and C-2 [24], mainly shared by Greenlanders and Danes. These clusters are associated with homelessness, alcohol abuse, and other social risk factors [24].

Migrants of "other origin" had the lowest proportion of clustered strains as expected, as the geographic origin in this pooled group of migrants is diverse and their time in DK short.

### Sex-age distribution

Compared to Danes, migrants in general presented with TB at an earlier age, reflecting early primary infection in their country of birth. IR of clustered TB distributed, as expected for high incidence countries, with an almost even overall sex-distribution, whereas Danes had around two to three times more males, higher at specific age groups, in accordance with NR for Danes [25]. Also, compared to other Scandinavian countries, DK has a high TB NR in middle-aged Danish males, particular in the larger cities, associated with transmission of C-2 [24].

A recent study [26] has documented a high rate of transmission in Inuit children in Greenland. This has not yet reflected in DK, as Inuit children and adolescence are strongly underrepresented. So are elderly Somalis and migrants of other origin.

### Control strategies

Micro epidemics and dominant clusters, like C-2, transmited among Danes, indicate that DK, being in a process aiming at TB elimination, still has unsolved TB control problems [27]. In the migrant population, the Somalis carry a major disease burden, mainly through reactivation disease. However, decreasing IR of clustered TB in this group is presently seen, following increased focus on the problem and longer time interval since infection. This may resemble the development observed for Vietnamese boat refugees arriving in DK [28], a previous high-risk group.

Active case finding and effective treatment of symptomatic individuals, especially in high risk groups, mainly from larger cities and often with homelessness and abuse problems, must be intensified for IR to decrease. Educating staff in high risk settings like shelters, prisons, clubs/associations for migrants, and schools, to recognize and inform about TB symptoms should be strengthened to increase active case finding. Also, preventive therapy to certain high risk groups, like persons co-infected with HIV, with fibrotic lesions, or with recent infection with MTB [29], can reduce the pool of latently infected persons.

Coordination of activities on national and regional level by experienced parties is crucial. Mapping of TB in migrants, e.g. by genotyping, may identify specific risk patterns within ethnic subgroups. This information is valuable for further targeting of TB control.

It is important that the population has continues free access to screening, diagnostics and treatment for TB, and this must be combined with active case finding in certain high-risk groups. E.g. migrants should not only be offered screening at arrival, as they have a continuous increased risk of developing TB many years post-migration [30]. However, the most important and cost-beneficial factor for TB control remains, especially for low incidence countries, investment in global TB control [31].

## Conclusion

In conclusion, based on continues nationwide genotyping from 1992 through 2004, we found only limited transmission between 1,780 Danes and 2,809 migrants in DK, representing 99% of all culture verified TB cases in the country during the 13 years study period. Fears that TB in migrants poses a threat for resident Danes seems exaggerated and unjustified, and we believe this to be true for other low incidence countries as well. TB-control efforts in DK need to focus on continues outbreaks and micro-epidemics in socially marginalized subpopulations, especially the Cluster-2 outbreak, a subtype primarily seen among Danish and Greenlandic males.

## Competing interests

The authors declare that they have no competing interests.

## Authors' contributions

ZKJ, ABA, AKJ, ICB, and TL have made substantial contributions to conception and design, acquisition of data, analysis and interpretation of data. MKJ, PHA, and VOT have been involved in drafting the manuscript and revising it critically for important intellectual content.

MKJ conducted all statistical analyses. All authors read and approved the final version of manuscript.

## Pre-publication history

The pre-publication history for this paper can be accessed here:

http://www.biomedcentral.com/1471-2334/12/60/prepub
